# Targeting mental health and wellbeing in women who have experienced gender-based violence through moderate-vigorous physical activity: a systematic review

**DOI:** 10.1186/s12966-025-01735-6

**Published:** 2025-04-24

**Authors:** Thea Baker, Niamh Mundell, Harriet Koorts, Michelle Pebole, Simon Rosenbaum, Elly Ganakas, Megan Teychenne

**Affiliations:** 1 Institute for Physical Activity and Nutrition (IPAN), School of Exercise and Nutrition Sciences, Deakin University, Geelong, Australia; 2 Translational Research Center for TBI and Stress Disorders National Network Center, VA Boston Healthcare System, Boston, MA USA; 3 Department of Psychiatry, Harvard Medical School, Boston, MA USA; 4 School of Clinical Medicine, Discipline of Psychiatry and Mental Health, University of New South Wales, Randwick, NSW 2031 Australia

**Keywords:** Gender-based violence, Intimate partner violence, Victim-survivors, Physical activity, Moderate-vigorous physical activity, Trauma-informed practice, Trauma-informed physical activity

## Abstract

**Background:**

Gender-based violence (GBV) is associated with high rates of psychopathology (i.e., depression, anxiety, post-traumatic stress disorder) in victim-survivors. Existing research has demonstrated that physical activity is beneficial for mental health and wellbeing across various populations. However, it is currently unclear whether moderate-vigorous physical activity (MVPA) is efficacious for victim-survivors of GBV. Therefore, this systematic review aims to understand 1) the acceptability and feasibility of leisure-time MVPA interventions for victim-survivors of GBV, 2) the efficacy of leisure-time MVPA interventions for mental health and wellbeing in this cohort, and 3) the implementation strategies used in the development of such interventions.

**Methods:**

Four databases were searched from inception to January 2024. Leisure-time MVPA intervention studies that reported on at least one measure of mental health or wellbeing for self-identified/biological women who had lived experience of GBV were eligible.

**Results:**

Eleven studies met inclusion criteria, and analysis revealed a range of different types of MVPA (*n* = 5) and mental health/wellbeing outcomes measured (*n* = 9). The main findings include: 1) feasibility and acceptability of MVPA for victim-survivors was enhanced where trauma and violence-informed (TVI) practices were used in the development and delivery of interventions. 2) There was a lack of clarity and consistency around TVI practice in physical activity intervention research. 3) Leisure-time MVPA may be positively associated with mental health and wellbeing.

**Conclusions:**

Limited evidence exists regarding the impact of MVPA on mental health and wellbeing for this important population group. Future studies should embed TVI strategy within the design, delivery, and implementation of interventions.

**Supplementary Information:**

The online version contains supplementary material available at 10.1186/s12966-025-01735-6.

## Background

Gender-based violence (GBV) is a pressing public health issue that encompasses a range of human rights violations directed at an individual because of their perceived gender [1, 2]. Victim-survivors of GBV can experience “physical, sexual or psychological harm or suffering” in the public or private sphere, and forms include sexual violence, intimate partner violence (IPV), domestic and family violence [3]. GBV is characterised by behaviours associated with control within relationships and inequality of gendered power, which disproportionately impact women, girls and populations within the LGBTIQA + (lesbian, gay, bisexual, transgender, intersex, queer, asexual and other sexually or gender diverse) community [4, 5]. One in three women have been subjected to some form of GBV in their lifetime [1], and almost 18% of ever-partnered women (15–49 years old) will have experienced IPV in the last 12 months [6], with 32% of women experiencing severe physical IPV (i.e., being assaulted with a fist or weapon, beaten or strangled) [7, 8].

Women exposed to GBV experience high rates of psychopathology including post-traumatic stress disorder (PTSD), anxiety, depression, and substance misuse [9–11]. IPV is also an under-recognised risk factor for cardiovascular disease (the leading cause of death in women globally), [4] with physical forms of IPV amplifying this risk [7]. Despite evidence suggesting the inter-connected nature of these health impacts, support for victim-survivors is often targeted towards mental health interventions [2, 12]. Psychological treatments typically include trauma-focused therapies (i.e., trauma-focused Cognitive Behavioural Therapy [CBT], Eye Movement Desensitisation and Reprocessing [EMDR]), alongside pharmacological support [13]. Whilst considered gold-standard interventions for PTSD, treatment efficacy is often impacted by a range of factors including high dropout rates, limited help-seeking behaviours due to mental health stigma, financial barriers to accessing therapy, and long waitlists [14, 15]. The lack of effective, accessible, and holistic approaches to GBV recovery results in a chronic perpetuation of ill-health for victim-survivors, leading to calls to explore alternative/adjunctive approaches [16].

Physical activity (PA) is defined as ‘any bodily movement produced by skeletal muscles that requires energy expenditure’ [17], and is a valid and effective intervention to support mental health and wellbeing [18]. When seeking to leverage the mental health benefits of PA, contextual factors (i.e., PA type, domain, physical/social environment, supervision/delivery) may be an important consideration in the design of PA interventions [18]. An extensive body of evidence also indicates that leisure-time PA (i.e., PA during free-time/active travel) confer mental health benefits whilst work-related and domestic PA might have a detrimental impact on mental health and wellbeing [19, 20]. Existing literature suggest that this is likely associated with the greater opportunities for enjoyment and autonomy in leisure-time physical activity, both of which are important to enhance the mental health benefits of physical activity [19]. PA is efficacious in reducing the symptoms of anxiety, depression and PTSD [21–23], all frequently experienced by victim-survivors of GBV. Whilst research suggests that women experience higher rates of PTSD than men, women are underrepresented in PA studies which makes acceptability (i.e., whether the intervention is considered appropriate, suitable and satisfactory) [24] and feasibility (i.e., understanding whether an intervention can be done) [25] of PA interventions for women challenging [26, 27]. Recent reviews exploring PA interventions with a focus on PTSD/trauma/GBV, suggest positive impacts on mental health and wellbeing for women who have experienced GBV [28–30], however most studies have focused on yoga and holistic movement practices (HMP’s) (i.e., Pilates, Tai chi, Qigong) for this population [28, 30]. Despite the demonstrated efficacy of HMP’s for mental health, certain elements (i.e., specific yoga poses) may be triggering for women who have experienced sexual violence [31, 32]. Evidence from a recent study suggest that this population of women have preferences for a diverse range of exercise types/modes [33]. Lastly, HMP’s often do not meet the PA guidelines which highlight the importance of both aerobic and strength PA to maximise physical and mental health [17]. This suggests the need to expand the evidence-base of PA interventions, including more vigorous forms of PA, that are acceptable and feasible for victim-survivors [33].

Within mental health research there is a significant gap between translating effective treatments developed within clinical settings into real-world mental health settings (i.e., implementation factors) [34]. Rigid protocols effect clinical adoption, and fidelity (i.e., the degree to which it is delivered as intended) is impacted by a lack of adherence, and clinicians report finding them hard to fit into standardised appointment times within community mental health/private practice settings [35–37]. Literature from the GBV and healthcare fields suggests that there are a range of implementation challenges associated with interventions developed for victim-survivors of GBV. A central consideration is how the complex intersection of the varied health impacts linked to GBV is navigated within a health and human services system that does not easily accommodate such complex, multifactorial needs [38–40]. In response, GBV researchers have strongly advocated for the use of consumer involvement (i.e., both lived experience ‘experts’ and other relevant party ‘experts’) in the codesign of interventions for victim-survivors [41, 42]. Collaborative approaches to research and intervention design can reduce the research-action gap, ensuring that programs are appropriate for participant needs, can be implemented effectively in practice, and are considered foundational to development of trauma-informed services [42, 43].

Trauma and violence-informed (TVI) care and gender sensitive practices are service delivery approaches which acknowledge the impact of trauma and promote physical and psychological safety [44]. These practices have evolved within the human services and healthcare sectors in acknowledgement of the risks of re-traumatisation that accompany help-seeking and are essential to creating safe services for victim-survivors of GBV [45–47]. TVI care can boost engagement, optimise participant outcomes, and increase satisfaction (i.e., acceptability), yet have not been well-defined or clearly utilised within PA research settings [28, 30]. GBV victim-survivors face unique challenges regarding safety, meaning exercise spaces are often difficult places to access [48]. A recent scoping review has stressed the importance of TVI practices to increase accessibility to and acceptability of moderate vigorous physical activity (MVPA) (i.e., physical activity performed above three METS) [49] interventions for women with PTSD [29]. The impact of utilising gender-sensitive and TVI practices on the acceptability, feasibility, and successful implementation of MVPA interventions are unexplored, suggesting the need to target these approaches to support GBV victim-survivors engaging in MVPA [32, 50, 51].

This systematic review aims to synthesise the current literature on leisure-time MVPA interventions targeting mental health and wellbeing outcomes for women who have experienced GBV. Specifically, the research questions addressed were:

1. How acceptable and feasible are leisure-time MVPA interventions for victim-survivors of GBV (including use of TVI/gender-sensitive practices)?
2. How efficacious are leisure-time MVPA interventions for this cohort on mental health and wellbeing?
3. What implementation strategies have been reported in the development of leisure-time MVPA interventions for women who have experienced GBV?

## Methods

**Fig. 1:**
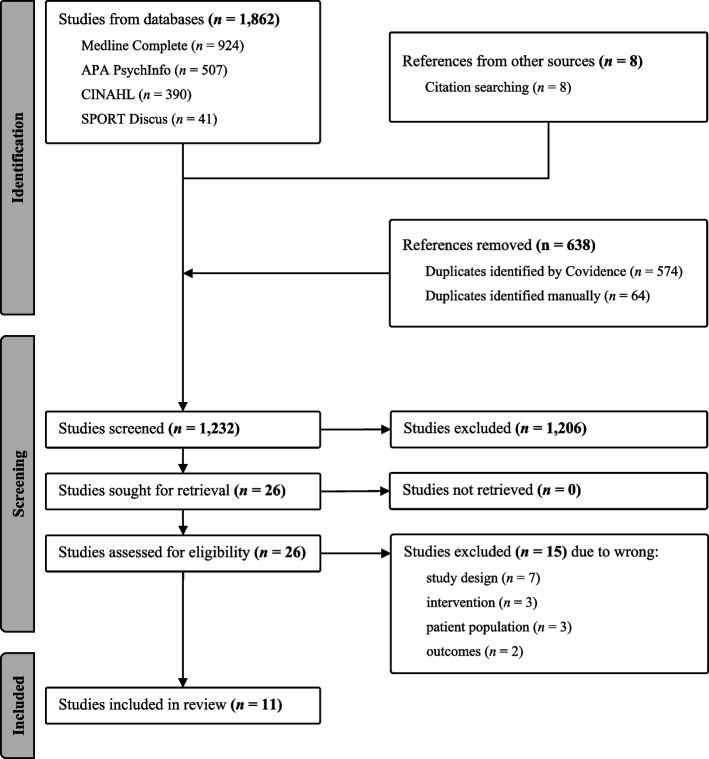
PRISMA flowchart of study selection

The protocol for this systematic review was registered on PROSPERO (record ID: CRD42023477921) and it followed the Preferred Reporting Items for Systematic Review and Meta-Analyses (PRISMA) guidelines (see Fig. 1) [52].

### Data sources and search strategy

Four academic databases (APA PsychInfo, CINAHL, Medline Complete and SPORTDiscus) were searched for papers from inception to January 3rd, 2024. Preliminary search concepts included women, violence, physical activity, and PTSD. The expanded selection of search terms utilised Medical Subject Headings (MeSH) and was informed by recent scoping reviews within this field [28, 29, 50], systematic reviews exploring physical activity, mental health and GBV [53, 54], and in consultation with a school research librarian. The complete search strategy is shown in Additional File 1.

### Eligibility criteria

Quantitative and qualitative studies (if anchored to an intervention) were evaluated against the following inclusion criteria: i) self-identified or biological women over the age of 18 who had lived experience of any direct form of GBV irrespective of the presence of a clinical diagnosis, ii) interventions that explored at least one form of leisure-time MVPA, including a broad range of aerobic and muscle-strengthening activities (see Additional File 1 for full details of search terms). Guided by a recent scoping review exploring MVPA for women who have a diagnosis of PTSD [29], the CDC’s ‘General Physical Activities by Level of Intensity’ [49] was used as a guide for decisions regarding level/type of intensity of physical activity, iii) outcomes included at least one validated measure of mental health or wellbeing (i.e., PTSD symptoms, anxiety symptoms, self-esteem, empowerment), iv) studies utilised standardised instruments, self-reported data and, in the case of qualitative studies, analyses from focus groups or individual interviews.

Studies were excluded if the form of MVPA was a holistic movement practice (i.e., yoga, Pilates, Tai chi or Qigong), or where English language translations or full text versions were unavailable. Grey literature, theses, protocols, policy statements, reviews and non-peer reviewed studies were also excluded. Where pertinent results data could not be gleaned within the text of the paper, the corresponding author of the study was contacted via email twice over four weeks for clarification prior to inclusion. If no response was received within this timeframe, or data were unable to be verified the paper was excluded. The full PICOS Framework is shown in Additional File 2.

### Data extraction

Studies that met the search criteria were uploaded into Endnote (v20.6), duplicates were removed, and remaining articles were uploaded to Covidence. Prior to initial title and abstract review, two researchers (TB, and EG blinded for review) independently conducted a pilot screen of 10 articles to establish consistency of approach. Following this initial pilot screening, two researchers (TB and EG) independently assessed titles and abstracts, and subsequently, full texts against the inclusion criteria. Disagreements were managed via a review meeting where final decisions were made regarding inclusion. To include all relevant sources reference list screening and forwards/backwards citation tracking was conducted at data extraction stage. The final list of studies included for full text screen were agreed at a team meeting with members of the research team (TB, NM, HK, SR and MT).

Data were extracted by two researchers (TB and EG) independently. Prior to completing data extraction for the full sample, a pilot extraction was conducted by the two researchers for a subset (*n* = 2) of randomly selected articles, with results compared for reliability. A range of data were extracted including study design information and population characteristics, and intervention contextual features (i.e., type of MVPA, physical environment, social environment and form of supervision/delivery (i.e. whether online or in-person) [18]. Acceptability (i.e., whether the intervention was considered appropriate, suitable, and satisfactory) was reported based on participant perspectives from focus groups and interviews, in addition to the use of TVI/gender-sensitive practices. These were extracted based on TVI principles of safety, trustworthiness/transparency, peer support, collaboration/mutuality, empowerment/voice/choice and cultural/historical/gender issues [47]. Feasibility was reported based on recruitment, retention, and compliance rates. Improvements in mental health and wellbeing outcomes (i.e., PTSD, depressive symptoms, resilience) were derived from interventions that reported statistically significant positive changes to mental health/wellbeing outcomes. Collectively these insights helped to determine the overall impact of an intervention on the mental health and wellbeing of participants. Lastly, study components that explored factors relating to the translation of interventions into real-world settings (i.e., fidelity, implementation strategies) were extracted [55]. Flay et al.’s description was used to differentiate between efficacy and effectiveness study aims for the purposes of data extraction [56].

### Data synthesis

Due to heterogeneity of study design, outcomes, and intervention approaches, conducting a meta-analysis was not appropriate. Instead, quantitative findings were synthesised narratively, following Cochrane guidelines [57], for qualitative studies key themes were identified and summarised in line with a thematic synthesis [58], and mixed methods studies contributed separately to both types of synthesis.

### Methodological quality

Study quality was assessed independently by two authors (TB and EG) using the Mixed Methods Appraisal Tool (MMAT) [59]. The MMAT was developed to support the assessment of multiple study designs within a single tool and is considered a reliable appraisal tool for mixed methods research [60, 61]. It includes two screening questions for all studies, followed by five questions per study design (quantitative, qualitative, mixed methods) with the response options: ‘yes’, ‘no’ or ‘can’t tell’. Authors of the MMAT discourage the creation of overall scores, as they argue that readers should be able to see which aspects of the study were included or not (see Additional File 3), however studies with greater ‘no’ or ‘can’t tell’ responses tend to be associated with lower methodological quality [59].

## Results

In total 1,862 publications were identified from the database search, and eight additional articles sourced from citation searches. Duplicates were removed (*n* = 638), leaving 1,232 for title and abstract screening. Twenty-six publications were deemed eligible for inclusion in full text screening (see Fig. 1).

### Study characteristics

Of the 11 eligible studies, 10 were published between 2017 and 2023, with the remaining study published in 2006. Six studies were conducted in the USA [62–67], two in Spain [68, 69], two in Canada [70, 71] and one in France [72]. With regards to the study design, three were non-randomised pilot studies [64, 66, 70], three were mixed methods studies (two combined a randomised controlled trial (RCT) with focus groups [63, 68] and the other included a feasibility study with focus groups [67]), two utilised qualitative methodologies only [69, 71], whilst the remaining three studies included an RCT [72], a pilot RCT [65] and a cross-sectional study which was embedded within a larger mixed-methods study [62]. Sample sizes ranged from *n* = 1 to *n* = 700, and 100% of participants across all studies were self-identified or biological women. The type of GBV experienced by participants included: sexual violence [62, 65], intimate partner violence (IPV) [63, 68], interpersonal victimisation [64], sexual victimization [67], GBV [70], domestic violence [72], military sexual trauma [66], male violence against women [69], and teen dating violence [71]. Three studies involved participants who had experienced GBV alongside women who had no lived experience of GBV [64, 65, 67]. Two of these studies reported results separately [64, 65]. The remaining study (*n* = 40) included two women (5% of sample) who had no lived experience of sexual victimisation [67]. Ten different mental health and wellbeing outcomes were measured across the nine quantitative studies including, PTSD (*n* = 5) [63–67], depressive symptoms (*n* = 3) [64, 66, 68], self-esteem (*n* = 3) [68, 70, 72], self-efficacy (*n* = 3) [66–68], generalised anxiety (*n* = 1) [64], empowerment (*n* = 1) [62], psychological distress (*n* = 1) [63], rumination (*n* = 1) [65], quality of life (which included 4 domains: physical, psychological, social and environmental) (*n* = 1) [70], and resilience (*n* = 1) [70].

### Contextual features of interventions

#### Type of PA

Three interventions utilised dance [63, 69, 71], three involved a form of self-defence [64, 66, 67], two explored boxing [62, 70], two delivered a mixed aerobic physical activity intervention (i.e., running, use of cardio-machines, aerobic classes) [65], and one involved a range of outdoor adventure activities [68].

#### Physical environment

Nine interventions were conducted indoors [62–67, 70–72], one multi-component mixed methods study was conducted outdoors in nature [68], and one study did not report on the physical environment [69]. See Table 1 for further details.

**Table 1:** Summary study characteristics

Author & year	Location	Study population	Study design	Type of GBV	MVPA intervention	Mental health/Wellbeing outcome	Physical environment	Social environment	Supervision/Support
Cole & Ullrich-French (2017) [62]	USA	*n* = 149, victim-survivors of sexual violence, aged between 18–69, majority identified as White (84%), most had completed tertiary education (63%)	Cross-sectional	Sexual violence	Boxing *Control:* group fitness classes	*Empowerment -*Empowerment in Exercise Scale [EES] (Moore & Fry, 2014)	*Indoors* – gym setting attached to university	*Group*	*Supervised* – qualified boxing instructor
Legrand & Crombez-Bequet (2022) [72]	France	*n* = 36, victim-survivors of domestic violence, mean age 33.4 30.6% had attended tertiary education	RCT	Domestic violence	Mixed MVPA *PA intervention:* 6 wks, 35–40 min 2 × p/wk *Control:* TAU	*Global Self-Esteem* – Self-esteem evaluation in the PA domain [ISP- 25] (Ninot et al., 2000)	*Indoors* – in participants homes	*Individual*	*Supervised* – sport science students
Shors et al. (2018) [65]	USA	*n* = 105 (*n* = 32*), women with and without history of sexual violence, aged between 18–32 (mean age 20)	Pilot RCT	Sexual violence	Moderate aerobic exercise *PA intervention:* 6 wks, 2 × 30 min p/wk. Treadmill/elliptical machines or aerobic exercise class *Control:* W/L	*Rumination* – Ruminative Responses Scale [RSS] (Treynor et al., 2003)	*Indoors* – exercise facility	*Group*	*Supervised* – trained aerobics instructors
Gammage et al. (2022 [70])	Canada	*n* = 56, victim-survivors of gender-based violence, aged between 18–66 (mean age 35), 41.2% identified as White/Caucasian, 78.4% had completed some level of tertiary education	Non-randomised pre-post	Gender-based violence	Boxing 14 wks, 1 × 90 min p/wk	*Quality of Life* – WHO Quality of Life measure [WHOQOL-BREF] (WHO, 1998) *Self-esteem* – Rosenberg Self-Esteem Scale [RSE] (Rosenberg, 1965) *Resilience* – Resilience Scale (Wagnild & Young, 1993)	*Indoors* – boxing studio within mixed-used gym space	*Group*	*Supervised* – qualified boxing coach *Mental health support* – social worker present
Holmes et al. (2021) [64]	USA	*n* = 82 (*n* = 49*), women with and without history of interpersonal victimisation, aged between 18–66 (mean age 33.1), 59% identified as White/European American, 69% had completed some form of tertiary education	Non-randomised pre-post	Inter-personal vicitim-isation	Rape Aggression Defence Course 4 × 3-h sessions over 4 days or 2 × p/wk for 2 weeks	*Generalised anxiety* - Generalised Anxiety Disorder–7 [GAD- 7] (Spitzer et al., 2006)	*Indoors* – university policy department	*Group*	*Supervised* – police officers
David et al. (2006) [66]	USA	*n* = 10, victim-survivors of military sexual trauma with PTSD, aged between 28–62 (mean age 48.3), 70% identified as non-Hispanic White, 40% had completed some tertiary education	Open trial pilot	Military sexual trauma	Self-defence and personal safety training 12 wks, 2 h 1 × p/wk	*PTSD—*Posttraumatic Stress Disorder Checklist for DSM- 4 [PCL-C] (Weathers et al., 1993) *Self-efficacy* – General Self-Efficacy Scale (Ozer & Bandura, 1990) *Depressive symptoms* – Beck Depression Inventory [BDI] (Beck et al., 1988)	*Indoors* – exercise facility	*Group*	*Supervised* – qualified martial artists *Mental health support* – psychologist present
Sáez et al. (2023) [68]	Spain	*n* = 34, victim-survivors of intimate partner violence, aged between 23–56 (mean age 40.75), 24.2% had completed tertiary education	Mixed methods (pilot RCT + focus groups)	Intimate partner violence	Adventure activities *Intervention group:* 8 wks, 3–5 h adventure activities p/wk *Control group:* TAU	*Self-esteem* – Rosenberg Self-Esteem Scale – Spanish version [RSE] (Rosenberg, 1965) *Self-efficacy* – General Self-Efficacy Scale (Baessler & Schwarzer, 1996) *Depressive symptoms* – Beck Depression Inventory 2 [BDI-II] (Beck et al., 1996)	*Outdoors –* varied blue and green spaces	*Group*	*Supervised* – experts in sports science for adventure components *Mental health support* – therapists present
Hotchkiss et al. (2022) [67]	USA	*n* = 40, 95% of whom were victim-survivors of sexual victimisation, aged between 18–48 (mean age 22.8), 52.5% identified as White, 72.5% were full time students	Mixed methods (feasibility pilot + focus groups)	Sexual victim-isation	Self-defence (within feminist framework) 8 sessions in total (6 = MVPA) 3 h, 1 × p/wk	*PTSD—*Posttraumatic Stress Disorder Checklist for DSM- 4 [PCL-C] (Weathers et al., 1993) *Interpersonal elf-efficacy* – Interpersonal domain from women’s self-efficacy in relation to self-defence tool (Ozer & Bandura, 1990)	*Indoors –* university counselling centre	*Group*	*Supervised* – Self-defence instructors *Mental health support* – Counsellors involved as participant mentors
Özümerzifon et al. (2022 [63])	USA	*n* = 45, victim-survivors of intimate partner violence, aged between 23–48, majority identified as Black, Indigenous, and People of Colour (+ 75%), 32% had some tertiary education	Mixed methods (RCT + focus groups)	Intimate partner violence	Creative dance/movement program *Intervention:* 6 wks, 2 × 90 min p/wk (60 min PA) *Control:* TAU	*PTSD -*Posttraumatic Stress Disorder Checklist for DSM- 5 [PCL- 5] (Weathers et al., 2015) *Psychological distress*—Kessler K- 6 Nonspecific Distress Scale [K- 6] (Kessler et al., 2003)	*Indoors* – non-public space	*Group* (virtual)	*Supervised* – two dance/movement facilitators
Iranzo-Domingo et al. (2022) [69]	Spain	*n* = 700, victim-survivors of male violence against women, aged between 18–70 (mean age 40)	Qualitative	Male violence against women	Dance movement therapy – ‘social dance’	NA	*Not stated*	*Group*	*Supervised* – dance therapists *Mental health support* – psychologist & social worker
Margolin (2019) [71]	Canada	*n* = 4 (*n* = 1*), a victim survivor of ongoing dating violence against adolescent women, aged 18	Qualitative – case study	Teen Dating Violence	Creative dance/movement 1 × p/wk for 3 months	NA	*Indoors* – dance studio within High School	*Group*	*Supervised* – author (and facilitator) a dancer and counsellor *Mental health support* – as above

^*^n = participants who had experienced GBV/were included in the study results reported

#### Social environment

All the studies were designed as group interventions, however due to COVID- 19 regulations, one RCT [72] had to be delivered individually.

#### Delivery/Supervision

All of the studies were supervised and delivered by instructors (see Table 1 for further details), and were delivered in person, with the exception of one mixed-methods study which moved to an online delivery mode during COVID- 19 [63]. Five studies (three mixed methods, one qualitative study and one non-randomised pilot) also included a qualified mental health support person who was present throughout MVPA sessions [63, 67–70].

### Acceptability and feasibility

Two studies included qualitative data providing insights into the feasibility and acceptability of the MVPA interventions [67, 68]. In terms of acceptability, participants involved in a multi-component self-defence intervention (*n* = 40) suggested that more time should be provided for group debriefing and development of self-care strategies as some scenarios were cited as “triggering”. [67] Both mixed methods studies [67, 68] highlighted the importance of group cohesion (i.e., being with other women victim-survivors with whom they had a shared experience of GBV) and staff support as playing a significant role in participant attendance and enthusiasm towards the intervention. In terms of feasibility, recruitment for a multi-component self-defence intervention (*n* = 40) was negatively impacted by the time commitment required by participants (26 h over 8 weeks) [67], whilst participants (*n* = 34) in a multi-component outdoor activity intervention felt the program was too short (24–40 h over 8 weeks) and ended too abruptly [68]. Forty-four percent of participants in this study also articulated that the distance/time to travel to the various outdoor activities was prohibitive [68].

Retention and attrition rates were reported in five of the nine quantitative studies, providing some insight into feasibility of the interventions [64, 66–68, 72]. Across these studies retention in the intervention was high, ranging from 85 to 100%. One mixed methods intervention (*n* = 45) [63] involved the option of attending an online focus group at the end of the intervention and authors reported that 64% of participants chose to join. Reasons for not joining were not reported. This study was also the only one to report compliance data [63]. Attendance across the 12 sessions was low, ranging from 1–11 sessions, with a median attendance of 5 sessions, 38% of participants attended < 5 sessions, and 27% participated in + 8 sessions [63]. Reasons for non-attendance to this virtual intervention included technical issues, distractions from children who were home-schooling and discomfort with engaging in a dance intervention virtually [63].

Intervention feasibility was also explored via study recruitment rates (i.e., expression of interest through to participation). One mixed-methods study (*n* = 45) reported on recruitment rates [63]. This dance intervention was developed with involvement from a community support organisation for victim-survivors of IPV, and recruitment was conducted via staff members. Of the 66 women who expressed initial interest in the study, 80% attended an orientation session, 70% provided informed consent and 68% went on to complete the study. “Scheduling issues” and “unknown reasons” were given for not participating [63].

### Trauma and violence-informed practice

Nine studies [62, 63, 65–71] included elements of trauma-informed practice (i.e., considerations around safety, trust, choice, collaboration), however only four studies [63, 67, 70, 71] articulated their approach to trauma-informed care, and none attempted to define trauma-informed PA. None of the studies explicitly used a TVI approach. Five studies included an additional mental health support person during PA sessions [63, 67–70], four studies had embedded trauma treatment frameworks within the design of the intervention [63, 67, 70, 71], four interventions included an element of group counselling/psychoeducation as part of their study design [66–69], whilst two other interventions incorporated group sharing/debrief time at the end of each MVPA session [62, 70], and three studies addressed trauma-informed training for facilitators/staff [62, 63, 70]. One non-randomised pilot study (*n* = 56) extensively listed the various trauma-informed practices, which included maintaining a consistent layout of the physical space to provide safety, and the use of a ‘door person’ to ensure that only those involved in the study could enter the gym space [70]. With respect to gender sensitive practice, only one intervention (12-week dance/movement program for IPV victim-survivors) was open to all gender identities, however all participants identified as women (*n* = 45) [63]. A 14-week boxing intervention included eligibility for participants who identified as women (cis or trans) [70], and an 8-week self-defence intervention was open to cisgender women [67].

### Impact on mental health and wellbeing

Of the nine quantitative studies, seven reported positive impacts on a range of mental health and wellbeing outcomes including PTSD, depressive symptoms, self-esteem, self-efficacy, empowerment, quality of life and resilience [62, 63, 66–68, 70, 72]. Of the five studies that explored PTSD two reported a reduction in PTSD symptoms [63, 67]. Two studies found no impact on PTSD symptoms (one pilot RCT [65], one non-randomised pilot study) [66], whilst another non-randomised pilot study reported PTSD severity did not improve at post-test but was reduced from baseline to 3-month and 6-month follow ups [64]. All three studies measuring self-esteem reported improvements (one RCT [72], one non-randomised pilot [70], one mixed methods) [68]. Three studies also reported mixed results for depressive symptoms, where two studies cited improvements (mixed methods [68] and non-randomised pilot) [66], whilst another non-randomised pilot reported no improvements [64]. Mixed results were reported for self-efficacy where two studies cited improvements (both mixed methods design) [67, 68], whilst a non-randomised pilot study reported no change [66]. Quality of life and resilience also improved according to results from one non-randomised pilot intervention [70], and higher rates of empowerment were reported in women who participated in the intervention attached to the cross-sectional study. [62] No improvements to psychological distress [63], generalised anxiety [64], and rumination [65] were reported.

Most of the quantitative studies (*n* = 5) collected data at baseline and post-intervention, however authors were often imprecise in reporting details of the final data collection period [63, 65, 67, 70, 72]. Three studies, two non-randomised pilot studies [64, 66] and one mixed methods study [68] included some follow-up assessment. Follow-up periods ranged from six weeks [64] to six months [66, 68]. One of the non-randomised pilot studies [64] only collected data at baseline (end of first session) and at the six-week post-intervention follow-up, making it hard to determine whether improvements were sustained. The other non-randomised pilot study [66] reported that improvements in depressive symptoms were sustained at both three and six months, whilst the remaining mixed methods study did not report the results of follow up data collection [68].

Data (from quantitative studies) reporting the impact of mental health and wellbeing outcomes explored through the lens of contextual factors (i.e., type of MVPA, physical environment, social environment, and supervision/delivery) are reported in Table 2.

**Table 2:** Change in mental health and wellbeing outcomes explored through lens of contextual factors

Contextual factor			Studies (*n*)	Mental health/wellbeing outcome	Improvement/No improvement
**Type of MVPA**	*Dance*		1	PTSD (*n* = 1) Psychological distress (*n* = 1)	✔ ×
	*Self-defence*		3	PTSD (*n* = 3) Self-efficacy (*n* = 2) Depressive symptoms (*n* = 2) Generalised anxiety (*n* = 1)	✔✔ × ✔ × ✔ × ×
	*Boxing*		2	Self-esteem (*n* = 1) Empowerment (*n* = 1) Quality of Life (*n* = 1) Resilience (*n* = 1)	✔ ✔ ✔ ✔
	*Mixed aerobic MVPA*		2	PTSD (*n* = 1) Self-esteem (*n* = 1) Rumination (*n* = 1)	× ✔ ×
	*Adventure activities*		1	Self-efficacy (*n* = 1) Self-esteem (*n* = 1) Depressive symptoms (*n* = 1)	✔ ✔ ✔
**Physical environment**	*Indoors*		8	PTSD (*n* = 5) Self-efficacy (*n* = 2) Self-esteem (*n* = 2) Depressive symptoms (*n* = 2) Empowerment (*n* = 1) Quality of Life (*n* = 1) Resilience (*n* = 1) Psychological distress (*n* = 1) Generalised anxiety (*n* = 1) Rumination (*n* = 1)	✔✔✔ × × ✔ × ✔✔ ✔ × ✔ ✔ ✔ × ×
	*Outdoors*		1	Self-efficacy (*n* = 1) Self-esteem (*n* = 1) Depressive symptoms (*n* = 1)	✔ ✔ ✔
**Social environment**		*Individual*	1	Self-esteem (*n* = 1)	✔
		*Group*	8	PTSD (*n* = 5) Self-efficacy (*n* = 3) Self-esteem (*n* = 2) Depressive symptoms (*n* = 3) Empowerment (*n* = 1) Quality of Life (*n* = 1) Resilience (*n* = 1) Psychological distress (*n* = 1) Generalised anxiety (*n* = 1) Rumination (*n* = 1)	✔✔✔ × × ✔✔ × ✔✔ ✔✔ × ✔ ✔ ✔ × × ×
**Delivery/Supervision**		*Unsupervised*	0	-	-
		*Trained/qualified instructor*	9	PTSD (*n* = 5) Self-efficacy (*n* = 3) Self-esteem (*n* = 3) Depressive symptoms (*n* = 3) Empowerment (*n* = 1) Quality of Life (*n* = 1) Resilience (*n* = 1) Psychological distress (*n* = 1) Generalised anxiety (*n* = 1) Rumination (*n* = 1)	✔✔✔ × × ✔✔ × ✔✔✔ ✔✔ × ✔ ✔ ✔ × × ×
		*Additional mental health support person*	4	PTSD (*n* = 2) Self-efficacy (*n* = 2) Self-esteem (*n* = 2) Depressive symptoms (*n* = 1) Quality of Life (*n* = 1) Resilience (*n* = 1) Psychological distress (*n* = 1)	✔✔ ✔✔ ✔✔ ✔ ✔ ✔ ×
		*No additional mental health support person*	5	PTSD (*n* = 3) Self-efficacy (*n* = 1) Self-esteem (*n* = 1) Depressive symptoms (*n* = 1) Empowerment (*n* = 1) Generalised anxiety (*n* = 1) Rumination (*n* = 1)	✔ × × × ✔ ✔ ✔ × ×

✔ = improvement reported/× = no improvement reported/*MVPA* Moderate vigorous physical activity/*PTSD* Post Traumatic Stress Disorder

### Implementation considerations

Whilst four studies explored established MVPA programs [62, 63, 66, 70], only one of these sought to measure effectiveness [70], and even fewer studies (*n* = 2) explored factors that might impact translation of MVPA interventions for this cohort into real-world settings [63, 67]. One mixed methods dance intervention (*n* = 45) [63] explored the feasibility of using an existing dance movement program, ‘Move to Move Beyond’ in a virtual format during COVID- 19. The intervention had been previously piloted with victim-survivors of IPV, and their feedback was integrated into the design of the present study (i.e., consumer involvement). Weekly post-workshop meetings were conducted where facilitators debriefed sessions and researchers tracked deviation from the curriculum to explore fidelity, however the authors do not report further on outcomes.

One mixed methods self-defence intervention (*n* = 40) explored the feasibility of the program within a university counselling setting with specific aims around implementation and evaluation [67]. They employed four implementation strategies to secure approval for long-term institutional support and funding: 1) building relationships with the program delivery team (established self-defence program), 2) involvement of the university as a stakeholder throughout the intervention, 3) promoting involvement and support within the counselling centre (also considered as stakeholders) and 4) securing long-term funding [67].

### Methodological quality

Quality assessment scores are reported in Additional File 3. In brief, most studies were of moderate/low methodological quality with most studies (*n* = 7) scoring more ‘no’ or ‘can’t tell’ for items beyond the screening questions, than they scored ‘yes’ for items [64–66, 68–70, 72].

## Discussion

This review identified 11 studies that evaluated the acceptability, feasibility, and efficacy/effectiveness of leisure-time MVPA interventions on the mental health and wellbeing of women who have experienced GBV. While previous scoping reviews have explored elements of MVPA for PTSD or trauma-informed approaches to PA, to the best of our knowledge this is the first systematic review exploring leisure-time MVPA interventions for women who have experienced any form of GBV [28, 29]. Results from this review broadly suggest that interventions aiming to increase leisure-time MVPA may be feasible and acceptable for this cohort, although few studies reported on implementation factors. In addition, leisure-time MVPA appears to be positively associated with a range of mental health and wellbeing outcomes (i.e., self-esteem, quality of life, resilience, and empowerment, with mixed results for self-efficacy and PTSD symptoms). Findings also underscore the importance of including TVI/gender sensitive practices for PA interventions among this population.

### Contextual features of interventions

There were positive impacts on mental health and wellbeing across the full range of different types of MVPA interventions included in this review (i.e., dance, self-defence, boxing, mixed aerobic physical activity, and outdoors adventure activities). This supports existing literature that suggests diverse types of physical activity positively impact mental health [73–76]. Thus, selection of physical activity should be informed by personal preference, enjoyment, and other contextual factors which have been shown to enhance the mental health benefits of physical activity [18]. Results from some of the qualitative data [63, 67, 68] highlight time as a barrier for participants, which is consistent with other research citing the high rates of domestic PA amongst women alongside family/caring responsibilities, emphasising the need to consider time-flexible approaches to interventions for population [77]. Existing research suggests that the physical environment can play an important role when seeking to leverage mental health benefits from physical activity [18] however improvements to mental health and wellbeing outcomes were reported in both indoor and outdoor environments from this review. As only one study was conducted in nature it is difficult to draw any specific conclusions regarding its importance for this population, however improvements were reported across all mental health and wellbeing outcomes measured (self-efficacy, self-esteem, depressive symptoms) [68].

Results suggest that the social environment may play an important role for victim-survivors of GBV. As only one intervention [72] was conducted individually (due to COVID- 19) it is hard to draw conclusions regarding the impact of individual interventions (vs group interventions) on mental health and wellbeing. However, participants stressed the importance of group cohesion and connection in providing a sense of collective support, safety, and motivation to engage in sessions, directly impacting retention rates, consistent with existing evidence from exercise with PTSD/sexual violence [33, 78]. The gender of participants (all identified as women) is likely to have enhanced this sense of safety and connection, a finding consistent with observational evidence elsewhere suggesting that women victim-survivors prefer women-only PA spaces and groups [31, 33]. The gender of instructors was not directly addressed in the design of the interventions in this review, however other literature has stressed the importance of involving female instructors for victim-survivors of GBV, underscoring the importance of gender-sensitivity for this community [31, 79]. All interventions were supervised by MVPA instructors; however, it is interesting that for this cohort of participants five studies [63, 67–70] included an additional mental health support person who was present throughout MVPA sessions. This supports recommendations highlighted in the literature regarding the importance of TVI practice to reduce barriers and increase participation in physical activity for this cohort [28, 29, 78] and perhaps suggests broadening the context of ‘supervision’ when working with particularly vulnerable populations [18].

### Trauma and violence-informed practice

This review highlights the importance of TVI practice for the development and implementation of interventions for women who have experienced GBV. All studies explicitly named a form of GBV within their study design, however one pilot RCT appeared to use the terms ‘sexual violence’ and ‘trauma’ interchangeably [65], and alongside two other interventions (one pilot study, one qualitative study) did not explicitly define the type of GBV [66, 67]. Without this clarity it is difficult to assess whether appropriate understanding and consideration has been given to the possible impact of the MVPA program on the lives of participants. Whilst 82% (*n* = 9) of studies [62, 63, 65–71] employed some aspects of trauma-informed practice, only four studies [63, 67, 70, 71] were intentional in specifying the need for trauma-informed care with victim-survivors and none of them explicitly used a more nuanced TVI approach. This aligns with calls within existing, albeit limited, research to develop the more intersectional, anti-oppressive approaches that are integrated into TVI practices, and to deploy these approaches at all stages of program design and delivery [28–30]. Consistent with evidence in clinical settings [44], where elements of trauma-informed practice were utilised, improvements to mental health and wellbeing outcomes (*n* = 7) were reported, with only one outcome (psychological distress) from one mixed methods study reporting no improvement [63]. By contrast, of the remaining studies that did not utilise trauma-informed practice, or applied it minimally, (*n* = 5) improvements to mental health and wellbeing were only reported from three studies [64–66, 69, 72].

The use of multi-component study designs (i.e., MVPA + group counselling) in four studies (two mixed methods, one non-randomised pilot and a qualitative study) supports creative approaches to developing safe spaces for victim-survivors to engage in physical activity [66–69]. While the results of this review support the use of multi-component study design for victim-survivors of GBV, it does make it challenging to determine the specific mechanisms that lead to improvements in mental health and wellbeing. This sentiment is supported in the broader GBV field, acknowledging that the complex psychosocial and physical health needs of victim-survivors often require multifactorial, interdisciplinary approaches [38, 39]. Whilst there is evidence elsewhere in the GBV/healthcare fields [41, 46, 80] that suggest these challenges can be supported via collaborative, codesign approaches to intervention research and development (also considered to be a foundational approach to TVI practice), there was little evidence of this across the studies in this review. One dance intervention (*n* = 45) had been previously piloted with victim-survivors of IPV, suggesting an element of consumer involvement in the current study design [63], and one self-defence intervention (*n* = 40) reported evaluation results from three types of relevant parties (‘stakeholders’) – participants, counsellors and the university (funding institution) [67]. Therefore, the results from this review support existing research that suggests TVI practice is an important consideration for feasibility, acceptability, and efficacy/effectiveness of MVPA interventions for women who have experienced GBV [28, 29, 32, 78].

## Limitations and future considerations

There are some limitations associated with this review. The small number of studies (*n* = 11) may limit the generalisability of results; however, this emphasises the need for more research in this field, echoed elsewhere in literature, particularly in more diverse contexts including immigrant/refugee communities and participants of colour [28–30, 78]. The intentional exclusion of studies which used HMP’s would have yielded a greater number of studies, however, may have detracted from an appreciation of how these other, under-researched forms of MVPA might play a role in the healing and recovery of women victim-survivors of GBV [29, 33]. Likewise, with its focus on women, this review cannot provide insights into how MVPA might support male, gender non-conforming and non-binary victim-survivors of GBV, which is an area for future research. Further, the overall low methodological quality of the interventions suggest results should not be overstated, again placing emphasis on the development of more, higher quality MVPA studies for this cohort. To better understand the cause-effect relationship, this might include the use of control groups, either in the form of RCT’s or a stepped-wedge design, which might be more ethical given the nature of the population [81, 82]. The results of the quality assessment also highlight the need to more accurately describe the PA measures, and their appropriateness in measuring the outcome, and to outline whether the intervention was delivered as intended. Factors influencing acceptability, feasibility and implementation factors were not well reported limiting conclusiveness of results. There were also a diverse range of mental health and wellbeing measures used (*n* = 10) impacting comparability of results. Future research might consider refining the focus of outcomes those that specifically target trauma-specific mental health and wellbeing, (i.e., PTSD, emotional dysregulation) most likely to be experienced by women who have experienced GBV. The GBV field is routinely challenged by the varied and sometimes confusing use of terminology. Across the 11 included studies, there were nine different forms of GBV, many of which were not clearly defined by authors. To aid interpretation and synthesis of data it would be helpful to utilise standardised terms and definitions across interventions. Lastly, whilst not a specific aim of this review, few studies explored either *physical activity outcomes* (one mixed methods study [63] attempted this however was impacted by virtual format of the intervention), or *physical health outcomes* (two studies explored this as part of self-report questionnaires) [63, 70]. Given the design of the interventions (i.e., MVPA) and the established comorbid physical health impact of GBV on victim-survivors it is perhaps a missed opportunity not exploring these outcomes and is a suggestion for future consideration.

## Conclusions

Leisure-time MVPA interventions may be acceptable and feasible for victim-survivors of GBV, and preliminary evidence suggests a positive association with mental health and wellbeing, which encourages the development and testing of future MVPA interventions for this population. This review also emphasises the importance of the development and application of a clearly defined, robust, and embedded TVI strategy, central to the design, implementation, and evaluation of the intervention. This should include exploring the preferences/needs of women with lived-experience as part of the intervention design (i.e., collaboration/codesign), TVI training for all team members involved in the study and perhaps developing additional components within the intervention to help participants translate their MVPA experience into their real-lives and recovery.

## Supplementary Information

Supplementary Material 1

Supplementary Material 2

Supplementary Material 3

## Data Availability

All data/search strings are included in this published article, and its supplementary information files.

## Abbreviations

CBT: Cognitive behavioural therapy
EMDR: Eye movement desensitisation and reprocessing
GBV: Gender-based violence
IPV: Intimate partner violence
HMP: Holistic movement practice
LGBTIQA +: Lesbian, gay, bisexual, transgender, intersex, queer, asexual and other sexually or gender diverse
MMAT: Mixed methods assessment tool
MVPA: Moderate Vigorous Physical Activity
PA: Physical Activity
PTSD: Post traumatic stress disorder
RCT: Randomised controlled trial
TVI: Trauma and violence-informed
