# Supplementary material for: Targeting mental health and wellbeing in women who have experienced gender-based violence through moderate-vigorous physical activity: a systematic review
Source: Int J Behav Nutr Phys Act. 2025 Apr 24;22:49. doi: 10.1186/s12966-025-01735-6 (PMC12023535; doi:10.1186/s12966-025-01735-6)
Supplement: Supplementary file 1 — Supplementary Material 1 [file 12966_2025_1735_MOESM1_ESM.docx]

*Additional File 1: Search strategy*

### Medline

|  | **Search terms** |
| --- | --- |
| **Concept 1 – keywords** | wom?n OR transwom?n OR female* |
| *OR* | |
| **Concept 1 – subject headings** | Women OR Female OR “Transgender Persons” |
| *AND* | |
| **Concept 2 – keywords** | violence OR “intimate partner violence” OR “domestic violence” OR “domestic abuse” OR “family violence” OR “family abuse” OR “sexual violence” OR “sexual abuse” OR “gender-based violence” OR “psychological violence” OR “psychological abuse” OR “financial violence” OR “financial abuse” OR “coercive control” |
| *OR* | |
| **Concept 2 – subject headings** | “Intimate Partner Violence” OR Rape OR “Dating Violence” |
| *AND* | |
| **Concept 3 – keywords** | “physical* activ*” OR exercis* OR danc* OR “self-defen?e” OR “martial arts” OR box* OR run* OR fitness OR sport* OR recreation* OR gym* OR “weight train*” OR “strength training” OR “resistance training” OR walk* OR cycl* OR bicycl* OR bik* |
| *OR* | |
| **Concept 3 – subject headings** | Exercise OR Running OR Sports OR “Martial Arts” OR Boxing OR Dancing |
| *AND* | |
| **Concept 4 - keywords** | “PTSD” OR rumination OR self-esteem OR empowerment OR resilience OR “social connectedness” OR “social support” OR anxiety OR depress* OR “self-efficacy” OR mastery OR “psychological distress” OR stress OR “quality of life” OR mood OR “mental health” OR “positive affect” OR “negative affect” OR “self-concept” OR “self-confidence” OR “mental illness” OR “mental disorder” OR wellbeing OR “well-being” |
| *OR* | |
| **Concept 4 –subject headings** | “Stress Disorders, Post-Traumatic” OR “Sexual Trauma” OR “Psychological Trauma” OR “Mental Health” OR Depression OR Anxiety OR “Self Efficacy” OR “Self Concept” OR “Psychological Distress” |

### CINAHL

|  | **Search terms** |
| --- | --- |
| **Concept 1 – keywords** | wom?n OR transwom?n OR female* |
| *OR* | |
| **Concept 1 – subject headings** | Women OR “Trans Women” |
| *AND* | |
| **Concept 2 – keywords** | violence OR “intimate partner violence” OR “domestic violence” OR “domestic abuse” OR “family violence” OR “family abuse” OR “sexual violence” OR “sexual abuse” OR “gender-based violence” OR “psychological violence” OR “psychological abuse” OR “financial violence” OR “financial abuse” OR “coercive control” |
| *OR* | |
| **Concept 2 – subject headings** | “Intimate Partner Violence” OR Rape |
| *AND* | |
| **Concept 3 – keywords** | “physical* activ*” OR exercis* OR danc* OR “self-defen?e” OR “martial arts” OR box* OR run* OR fitness OR sport* OR recreation* OR gym* OR “weight train*” OR “strength training” OR “resistance training” OR walk* OR cycl* OR bicycl* OR bik* |
| *OR* | |
| **Concept 3 –subject headings** | “Physical Activity” OR Sports OR Dancing OR Exercise OR “Physical Fitness” |
| *AND* | |
| **Concept 4 – keywords** | “PTSD” OR rumination OR self-esteem OR empowerment OR resilience OR “social connectedness” OR “social support” OR anxiety OR depress* OR “self-efficacy” OR mastery OR “psychological distress” OR stress OR “quality of life” OR mood OR “mental health” OR “positive affect” OR “negative affect” OR “self-concept” OR “self-confidence” OR “mental illness” OR “mental disorder” OR wellbeing OR “well-being” |
| *OR* | |
| **Concept 4 – subject headings** | “Stress Disorders, Post-Traumatic” OR “Mental Health” OR “Stress, Psychological” OR Depression OR Anxiety OR “Self-Efficacy” OR “Self Concept” OR Empowerment OR “Quality of Life” OR “Psychological Well-Being” OR Affect OR Hardiness |

### APA PsychInfo

|  | **Search terms** |
| --- | --- |
| **Concept 1 – keywords** | wom?n OR transwom?n OR female* |
| *OR* | |
| **Concept 1 – subject headings** | “Human Females” OR “Battered Females” |
| *AND* | |
| **Concept 2 – keywords** | violence OR “intimate partner violence” OR “domestic violence” OR “domestic abuse” OR “family violence” OR “family abuse” OR “sexual violence” OR “sexual abuse” OR “gender-based violence” OR “psychological violence” OR “psychological abuse” OR “financial violence” OR “financial abuse” OR “coercive control” |
| *OR* | |
| **Concept 2 – subject headings** | “Intimate Partner Violence” OR “Sexual Violence” OR “Dating Violence” OR “Domestic Violence” OR Rape OR “Acquaintance Rape” OR “Emotional Abuse” OR “Physical Abuse” OR “Verbal Abuse” |
| *AND* | |
| **Concept 3 – keywords** | “physical* activ*” OR exercis* OR danc* OR “self-defen?e” OR “martial arts” OR box* OR run* OR fitness OR sport* OR recreation* OR gym* OR “weight train*” OR “strength training” OR “resistance training” OR walk* OR cycl* OR bicycl* OR bik* |
| *OR* | |
| **Concept 3 –subject headings** | “Physical Activity” OR “Aerobic Exercise” OR “Physical Fitness” OR Exercise OR Sports OR “Martial Arts” OR Dance |
| *AND* | |
| **Concept 4 - keywords** | “PTSD” OR rumination OR self-esteem OR empowerment OR resilience OR “social connectedness” OR “social support” OR anxiety OR depress* OR “self-efficacy” OR mastery OR “psychological distress” OR stress OR “quality of life” OR mood OR “mental health” OR “positive affect” OR “negative affect” OR “self-concept” OR “self-confidence” OR “mental illness” OR “mental disorder” OR wellbeing OR “well-being” |
| *OR* | |
| **Concept 4 – subject headings** | “Posttraumatic Stress Disorder” OR “Posttraumatic Stress “OR “Stress and Trauma Related Disorders” OR “Emotional Trauma” OR Anxiety OR “Depression (Emotion)” OR “Major Depression” OR “Affective Disorders” OR “Well Being” OR “Quality of Life” OR “Mental Health” OR “Resilience (Psychological)” OR Distress |

### SPORTDiscus

|  | **Search terms** |
| --- | --- |
| **Concept 1 – keywords** | wom?n OR transwom?n OR female* |
| *OR* | |
| **Concept 1 – subject headings** | Women OR “TRANSGENDER athletes” |
| *AND* | |
| **Concept 2 – keywords** | violence OR “intimate partner violence” OR “domestic violence” OR “domestic abuse” OR “family violence” OR “family abuse” OR “sexual violence” OR “sexual abuse” OR “gender-based violence” OR “psychological violence” OR “psychological abuse” OR “financial violence” OR “financial abuse” OR “coercive control” |
| *OR* | |
| **Concept 2 – subject headings** |  |
| *AND* | |
| **Concept 3 – keywords** | “physical* activ*” OR exercis* OR danc* OR “self-defen?e” OR “martial arts” OR box* OR run* OR fitness OR sport* OR recreation* OR gym* OR “weight train*” OR “strength training” OR “resistance training” OR walk* OR cycl* OR bicycl* OR bik* |
| *OR* | |
| **Concept 3 – subject headings** | “PHYSICAL activity” OR EXERCISE OR “MARTIAL arts” OR “WOMEN’s sports” OR “WOMEN’s boxing” OR BOXING OR DANCE |
| *AND* | |
| **Concept 4 - keywords** | “PTSD” OR rumination OR self-esteem OR empowerment OR resilience OR “social connectedness” OR “social support” OR anxiety OR depress* OR “self-efficacy” OR mastery OR “psychological distress” OR stress OR “quality of life” OR mood OR “mental health” OR “positive affect” OR “negative affect” OR “self-concept” OR “self-confidence” OR “mental illness” OR “mental disorder” OR wellbeing OR “well-being” |
| *OR* | |
| **Concept 4 – subject headings** | “POST-traumatic stress disorder” OR “MENTAL health” OR ANXIETY OR “PEOPLE with mental illness” OR “MENTAL depression” OR “SELF-efficacy” OR “SELF-esteem” OR “SELF-perception” OR “WELL-being” OR “PSYCHOLOGICAL distress” OR “QUALITY of life” |
