# Supplementary material for: Targeting mental health and wellbeing in women who have experienced gender-based violence through moderate-vigorous physical activity: a systematic review
Source: Int J Behav Nutr Phys Act. 2025 Apr 24;22:49. doi: 10.1186/s12966-025-01735-6 (PMC12023535; doi:10.1186/s12966-025-01735-6)
Supplement: Supplementary file 2 — Supplementary Material 2 [file 12966_2025_1735_MOESM2_ESM.docx]

*Additional File 2: PICOS Framework for systematic search*

| PICOS Framework | Eligibility criteria | |
| --- | --- | --- |
|  | **Inclusion** | **Exclusion** |
| **P** (patient / population) | Self-identified / biological women >18 years of age  Experiences of GBV across the full spectrum including domestic violence, family violence, sexual violence, intimate partner violence  Women with clinical diagnoses of PTSD / other mental health conditions and those without formal diagnoses | <18 years of age, self-identified men / biological males, or gender-neutral individuals. |
| **I** (intervention / exposure) | MVPA interventions undertaken in the leisure-time domain  Individual / group interventions | HMP interventions (i.e., yoga, Pilates, Qi Gong, Tai Chi)  PA undertaken within the domains of transport (i.e., travel to/from work), domestic (i.e., household chores/gardening) or occupation (i.e., as part of job) |
| **C** (comparison / control) | Studies that include comparisons of no intervention, alternative form of PA including Mind-body interventions, treatment as usual or waitlist control  Where qualitative studies are included there may be no comparison condition |  |
| **O** (outcome) | At least one measure of mental health related outcome (i.e., PTSD symptoms, rumination, empowerment, self-esteem, resilience, social support/connectedness) |  |
| **S** (study design) | Qualitative and quantitative full-text articles assessing any MVPA interventions for women who have experienced GBV, which include standardised instruments, self-reporting and analysis from focus groups or individual interviews in the case of qualitative studies | Systematic reviews, narrative reviews |

*Appendix 3: MMAT Quality assessment results*

| **Study** | **Screening questions** | | **Qualitative** | | | | | **Quantitative RCTs** | | | | | **Quantitative non-randomized** | | | | | **Quantitative descriptive** | | | | | **Mixed methods** | | | | |
| --- | --- | --- | --- | --- | --- | --- | --- | --- | --- | --- | --- | --- | --- | --- | --- | --- | --- | --- | --- | --- | --- | --- | --- | --- | --- | --- | --- |
|  | *S1* | *S2* | *1.1* | *1.2* | *1.3* | *1.4* | *1.5* | *2.1* | *2.2* | *2.3* | *2.4* | *2.5* | *3.1* | *3.2* | *3.3* | *3.4* | *3.5* | *4.1* | *4.2* | *4.3* | *4.4* | *4.5* | *5.1* | *5.2* | *5.3* | *5.4* | *5.5* |
| Cole & Ullrich | Y | Y |  |  |  |  |  |  |  |  |  |  | N | Y | Y | Y | C/T |  |  |  |  |  |  |  |  |  |  |
| Özümerzifon et al. | Y | Y |  |  |  |  |  |  |  |  |  |  |  |  |  |  | C/T |  |  |  |  |  | Y | N | Y | Y | N |
| Holmes et al. | Y | Y |  |  |  |  |  |  |  |  |  |  | N | N | Y | Y | C/T |  |  |  |  |  |  |  |  |  |  |
| Shors et al. | Y | Y |  |  |  |  |  | C/T | C/T | C/T | C/T | C/T |  |  |  |  | C/T |  |  |  |  |  |  |  |  |  |  |
| Sáez et al. | Y | Y |  |  |  |  |  |  |  |  |  |  |  |  |  |  | C/T |  |  |  |  |  | N | N | N | C/T | N |
| Gammage et al. | Y | Y |  |  |  |  |  |  |  |  |  |  | N | N | N | C/T | C/T |  |  |  |  |  |  |  |  |  |  |
| Legrand et al. | Y | Y |  |  |  |  |  | N | Y | Y | C/T | N |  |  |  |  | C/T |  |  |  |  |  |  |  |  |  |  |
| David et al. | Y | Y |  |  |  |  |  |  |  |  |  |  | N | N | Y | N | C/T |  |  |  |  |  |  |  |  |  |  |
| Iranzo-Domingo et al. | Y | N | N | N | N | C/T | C/T |  |  |  |  |  |  |  |  |  |  |  |  |  |  |  |  |  |  |  |  |
| Margolin | Y | Y | Y | Y | C/T | Y | Y |  |  |  |  |  |  |  |  |  |  |  |  |  |  |  |  |  |  |  |  |
| Hotchkiss et al. | Y | Y |  |  |  |  |  |  |  |  |  |  |  |  |  |  |  |  |  |  |  |  | Y | Y | Y | Y | N |
