# Supplementary material for: Targeting mental health and wellbeing in women who have experienced gender-based violence through moderate-vigorous physical activity: a systematic review
Source: Int J Behav Nutr Phys Act. 2025 Apr 24;22:49. doi: 10.1186/s12966-025-01735-6 (PMC12023535; doi:10.1186/s12966-025-01735-6)
Supplement: Supplementary file 3 — Supplementary Material 3 [file 12966_2025_1735_MOESM3_ESM.docx]

*Additional File 3: MMAT Quality assessment results*

| **Study** | **Screening questions** | | **Qualitative** | | | | | **Quantitative RCTs** | | | | | **Quantitative non-randomized** | | | | | **Quantitative descriptive** | | | | | **Mixed methods** | | | | |
| --- | --- | --- | --- | --- | --- | --- | --- | --- | --- | --- | --- | --- | --- | --- | --- | --- | --- | --- | --- | --- | --- | --- | --- | --- | --- | --- | --- |
|  | *S1* | *S2* | *1.1* | *1.2* | *1.3* | *1.4* | *1.5* | *2.1* | *2.2* | *2.3* | *2.4* | *2.5* | *3.1* | *3.2* | *3.3* | *3.4* | *3.5* | *4.1* | *4.2* | *4.3* | *4.4* | *4.5* | *5.1* | *5.2* | *5.3* | *5.4* | *5.5* |
| Cole & Ullrich | Y | Y |  |  |  |  |  |  |  |  |  |  | N | Y | Y | Y | C/T |  |  |  |  |  |  |  |  |  |  |
| Özümerzifon et al. | Y | Y |  |  |  |  |  |  |  |  |  |  |  |  |  |  | C/T |  |  |  |  |  | Y | N | Y | Y | N |
| Holmes et al. | Y | Y |  |  |  |  |  |  |  |  |  |  | N | N | Y | Y | C/T |  |  |  |  |  |  |  |  |  |  |
| Shors et al. | Y | Y |  |  |  |  |  | C/T | C/T | C/T | C/T | C/T |  |  |  |  | C/T |  |  |  |  |  |  |  |  |  |  |
| Sáez et al. | Y | Y |  |  |  |  |  |  |  |  |  |  |  |  |  |  | C/T |  |  |  |  |  | N | N | N | C/T | N |
| Gammage et al. | Y | Y |  |  |  |  |  |  |  |  |  |  | N | N | N | C/T | C/T |  |  |  |  |  |  |  |  |  |  |
| Legrand et al. | Y | Y |  |  |  |  |  | N | Y | Y | C/T | N |  |  |  |  | C/T |  |  |  |  |  |  |  |  |  |  |
| David et al. | Y | Y |  |  |  |  |  |  |  |  |  |  | N | N | Y | N | C/T |  |  |  |  |  |  |  |  |  |  |
| Iranzo-Domingo et al. | Y | N | N | N | N | C/T | C/T |  |  |  |  |  |  |  |  |  |  |  |  |  |  |  |  |  |  |  |  |
| Margolin | Y | Y | Y | Y | C/T | Y | Y |  |  |  |  |  |  |  |  |  |  |  |  |  |  |  |  |  |  |  |  |
| Hotchkiss et al. | Y | Y |  |  |  |  |  |  |  |  |  |  |  |  |  |  |  |  |  |  |  |  | Y | Y | Y | Y | N |

*Key:* *S1. Are there clear research questions?* *S2. Do the collected data allow to address the research questions? Qual: 1.1 Is the qualitative approach appropriate to answer the research question?* *1.2 Are the qualitative data collection methods adequate to address the research question? 1.3 Are the findings adequately derived from the data? 1.4 Is the interpretation of results sufficiently substantiated by data? 1.5 Is there coherence between qualitative data sources, collection, analysis and interpretation? RCT’s: 2.1 Is randomization appropriately performed? 2.2 Are the groups comparable at baseline? 2.3 Are there complete outcome data? 2.4 Are outcome assessors blinded to the intervention provided? 2.5 Did the participants adhere to the assigned intervention? Non-randomised: 3.1 Are the participants representative of the target population? 3.2 Are measurements appropriate regarding both the outcome and intervention (or exposure)? 3.3 Are there complete outcome data? 3.4 Are the confounders accounted for in the design and analysis? 3.5 During the study period, is the intervention administered (or exposure occurred) as intended? Mixed methods:* *5.1 Is there an adequate rationale for using a mixed methods design to address the research question? 5.2 Are the different components of the study effectively integrated to answer the research question? 5.3 Are the outputs of the integration of qualitative and quantitative components adequately interpreted? 5.4 Are divergences and inconsistencies between quantitative and qualitative results adequately addressed? 5.5 Do the different components of the study adhere to the quality criteria of each tradition of the methods involved?*
